# The compositional and nano-structural basis of fracture healing in healthy and osteoporotic bone

**DOI:** 10.1038/s41598-018-19296-z

**Published:** 2018-01-25

**Authors:** Neashan Mathavan, Mikael J. Turunen, Manuel Guizar-Sicairos, Martin Bech, Florian Schaff, Magnus Tägil, Hanna Isaksson

**Affiliations:** 1 Department of Biomedical Engineering, Lund University, Lund, Sweden; 2 Department of Applied Physics, University of Eastern Finland, Kuopio, Finland; 3 Paul Scherrer Institut, Villigen PSI, Villigen, Switzerland; 4 Department of Medical Radiation Physics, Lund University, Lund, Sweden; 5 Lehrstuhl für Biomedizinische Physik, Physik-Department & Institut für Medizintechnik, Technische Universität München, Garching, Germany; 6 Department of Orthopaedics, Clinical Sciences, Lund University and Skåne University Hospital, Lund, Sweden

## Abstract

Osteoporosis, a prevalent metabolic bone disorder, predisposes individuals to increased susceptibility to fractures. It is also, somewhat controversially, thought to delay or impair the regenerative response. Using high-resolution Fourier-transform infrared spectroscopy and small/wide-angle X-ray scattering we sought to answer the following questions: Does the molecular composition and the nano-structure in the newly regenerated bone differ between healthy and osteoporotic environments? And how do pharmacological treatments, such as bone morphogenetic protein 7 (BMP-7) alone or synergistically combined with zoledronate (ZA), alter callus composition and nano-structure in such environments? Cumulatively, on the basis of compositional and nano-structural characterizations of newly formed bone in an open-osteotomy rat model, the healing response in untreated healthy and ovariectomy-induced osteoporotic environments was fundamentally the same. However, the BMP-7 induced osteogenic response resulted in greater heterogeneity in the nano-structural crystal dimensions and this effect was more pronounced with osteoporosis. ZA mitigated the effects of the upregulated catabolism induced by both BMP-7 and an osteoporotic bone environment. The findings contribute to our understanding of how the repair processes in healthy and osteoporotic bone differ in both untreated and treated contexts and the data presented represents the most comprehensive study of fracture healing at the nanoscale undertaken to date.

## Introduction

Skeletal regeneration following fracture is an intrinsically orchestrated cascade of cellular and molecular events culminating in reconstitution of the injury site and restoration of function^1–3^. The signalling network of these regenerative cascades also optimally coordinate the interplay between bone formation and bone resorption to ensure the structural and compositional integrity of the repaired bone is optimized to resist future fractures.

Osteoporosis, a common skeletal disease, is a condition which exhibits a pathophysiology characterized by the progressive and irreversible deterioration of the structural and compositional integrity of bone^4^. The pathogenesis of osteoporosis is distinguished by an imbalance in the bone remodelling process which favours bone resorption over bone formation resulting in a net loss of bone and subsequent increased bone fragility. The adverse impact of an osteoporotic bone environment on fracture healing remains controversial. Reviews of the literature conclude that while some evidence is suggestive of osteoporotic fractures representing a greater challenge to skeletal regeneration, a definitive consensus is absent^5,6^.

Irrespective of the underlying condition of the bone, delayed unions or recalcitrant non-unions necessitate interventions to stimulate an osteogenic response. Pharmaceutical interventions can manipulate the anabolic and catabolic responses during fracture repair to achieve a more favourable outcome^7^. Bone morphogenetic proteins (BMPs) promote osteogenesis through the differentiation of mesenchymal cells into osteoblasts and when applied as a stimulus BMPs have demonstrated faster bone formation and formation of larger bone volumes in challenging atrophic non-unions^8^. The therapeutic potential of BMPs as powerful anabolic agents has been extensively studied with two forms, BMP-2 and BMP-7, introduced clinically^9^. Cells of the osteoblastic lineage and of the osteoclastic lineage are intrinsically coupled and consequently BMPs are now recognized as powerful agonists in not only upregulating osteoblastogenesis but also upregulating osteoclastogenesis and osteoclast survival by direct stimulation of osteoclastic resorption or indirectly via the RANKL/RANK pathway^10^. To optimize the potential of BMPs, an anabolic–anti-catabolic paradigm has been explored to uncouple its effects in inducing premature bone and callus resorption^11,12^. Bisphosphonates, such as zoledronate (ZA), are anti-resorptive agents primarily established in the treatment of osteoporosis to retain bone mass through the promotion of osteoclast apoptosis^13^. In adopting this anabolic – anti-catabolic paradigm, the specific combination of BMP-7 with systemic ZA has proven highly effective with remarkably enlarged calluses and increased mechanical strength reported in animal models^7,11,12^.

At the nanoscale, the constituent components of bone consist of a soft collagenous phase and a stiff inorganic mineral phase. Collagen confers bone with its elastic properties to absorb energy during mechanical loading. Mineral, in the form of carbonated hydroxyapatite crystals, contributes to stiffness and compressive strength at the expense of flexibility. These constituents together form the various hierarchical levels that contribute to bone’s resistance to fracture^14^. Thus, bone composition and nano-structure are important determinants of its strength and the quality of the newly-formed bone following fracture is of significant interest in both healthy and osteoporotic contexts, as well as when the regenerative response is perturbed by pharmaceutical interventions. Moreover, quantifying heterogeneity in the spatial distribution of compositional and nano-structural constituents is integral to understanding the macroscopic mechanical response^15,16^. Vibrational spectroscopic techniques such as Fourier transform infrared (FTIR) spectroscopy and X-ray scattering techniques such as small and wide angle X-ray scattering (SAXS/WAXS) are established techniques in the characterization of bone material composition and nano-structure respectively^17–19^. Bone composition is probed with FTIR spectroscopy by interpreting structure-sensitive molecular vibrations by means of a series of validated spectral parameters to map the spatial distribution of physicochemical properties. The scattering patterns of SAXS/WAXS are sensitive to the electron density contrast between the mineral crystals and the organic matrix of bone, and the intensity distribution around the incident beam yields information on crystal dimensions and orientations within the illuminated bone volume.

Characterization of callus composition in healthy, untreated fracture models undergoing healing has previously been reported^20–22^. Yet compositional distinctions in the regenerative process in an osteoporotic environment or when subject to BMP or bisphosphonate treatments remain less well-characterized^23,24^. Furthermore, the literature lacks a comprehensive, quantitative analysis of bone healing at the nanoscale. Indeed the literature yields only few publications and these have reported qualitative characterizations based on sample sizes of 1–2^23,25,26^. This is understandable since access to high-resolution synchrotron-based measurement facilities is limited. Thus, an important distinction of this study is the characterization of the nano-structure at high resolution in numbers sufficient for statistical analysis. Moreover, fracture repair at the nanoscale in an osteoporotic bone environment has yet to be investigated.

In a previous publication, we reported radiological, micro-structural and mechanical assessments of healing in an open-fracture rat model to be equivalent in healthy and osteoporotic bone environments^6^. When subject to BMP-7 treatment however the osteogenic response appeared to differ. In healthy rats, radiological, microstructural and mechanical measures suggested a reduced osteogenic response compared to previous publications in young, male rats treated with BMP-7^11,12^. Thus, we speculated on the existence of an age or gender dependency of BMP-7. In contrast to the healthy rats, BMP-7 only treated osteoporotic fractures achieved 100% union and significantly increased measures of mineralized bone volume, total callus volume, peak force and absorbed energy in comparison to untreated osteoporotic fractures. Furthermore, BMP-7 induced total callus volumes were twice as large in OVX rats compared to Control rats. These findings are suggestive of an increased sensitivity of BMP-7 to an estrogen-deficient environment.

In the present study, our aim is to extend the investigation of these samples to consider if material composition and nano-structure characterizations support our previously reported findings. Specifically, our objective is to address the following questions: Are measures of material composition and nano-structure in newly-formed callus tissue during fracture repair equivalent in healthy and osteoporotic bone environments? And do interventions with BMP-7 alone or when synergistically combined with ZA differ in healthy and osteoporotic fracture repair?

## Materials and Methods

### Samples

Sham-operations (Control, n = 24) or ovariectomies (OVX, n = 25) were performed on 12-week old female Sprague-Dawley rats (Charles River, Germany). In the 18 weeks following ovariectomy, we have previously documented the presence of extensive microstructural deterioration characteristic of post-menopausal osteoporosis at multiple anatomical sites in this model^27^. Mid-diaphyseal osteotomies of the right femur were established at 24 weeks of age in all rats following the protocol of a well-described, open-fracture model of recalcitrant non-union^6,28^. In previous publications with this model, approximately 50% of fractures consistently result in non-unions at the six-week time point in young healthy male rats^6,12^. Subsequently, the rats were randomly allocated to one of six treatment groups: (i) Control (Untreated), (ii) OVX (Untreated), (iii) Control (BMP-7), (iv) OVX (BMP-7), (v) Control (BMP-7 + ZA) and (vi) OVX (BMP-7 + ZA). The details of the experimental surgeries have been previously reported^6^. BMP-7 (Osigraft, Stryker Biotech, Malmö, Sweden) was administered locally in 50 μg doses in the form of a putty placed circumferentially around the fracture site. The 50 μg dosage in rats is equivalent to the maximum dose recommended in humans on a milligram per kilogram basis^11^. At 2 weeks post-fracture, a single subcutaneous injection of either saline or systemic ZA (0.1 mg/kg) (Zometa, Novartis, North Ryde, NSW, Australia) was administered. On a milligram to kilogram basis, the doses in rats is approximately equivalent to a once yearly dose received by humans for the treatment of osteoporosis^11^.

Animals were housed in pairs under standard conditions with *ad libitum* access to food and water, and permitted unrestricted weight bearing. All experimental procedures and laboratory animal care were approved by the regional animal ethics and scientific advisory committee and in accordance with relevant national guidelines and regulations (Ethical Committee of Malmö/Lund, Ethical Permission No. M 316 - 11).

Six weeks following osteotomy (corresponding to 30 weeks of age), the rats were euthanized and the fractured femurs were harvested, Directly following harvest, radiological, micro-structural and whole bone mechanical properties were measured and have been reported separately^6^. Subsequently, the bones were dehydrated and embedded in epoxy resin. Bone composition and nano-structure of the fracture site were evaluated using FTIR spectroscopy and SAXS/WAXS respectively.

### Fourier-transform infrared (FTIR) spectroscopy

Longitudinal sections (4 µm thick) of the epoxy-embedded femurs were sectioned using a HM 355S microtome (Thermo Scientific, MA, USA) and transferred onto barium fluoride (BaF_2_) windows. Spectral acquisition in transmission mode was performed at the D7 beamline of the MAX-IV laboratory (Lund, Sweden) using a Bruker 66V FTIR spectrometer coupled to a Bruker Hyperion 3000 IR microscope. Spectral acquisition spanned 2000 cm^−1^ to 800 cm^−1^ using a spectral resolution of 4 cm^−1^ and 64 repeated scans. A focal plane array (FPA) detector covering 341 x 341 µm divided into 128 x 128 elements was used, which after binning resulted in a spatial resolution of 5.3 µm.

**Figure 1:**
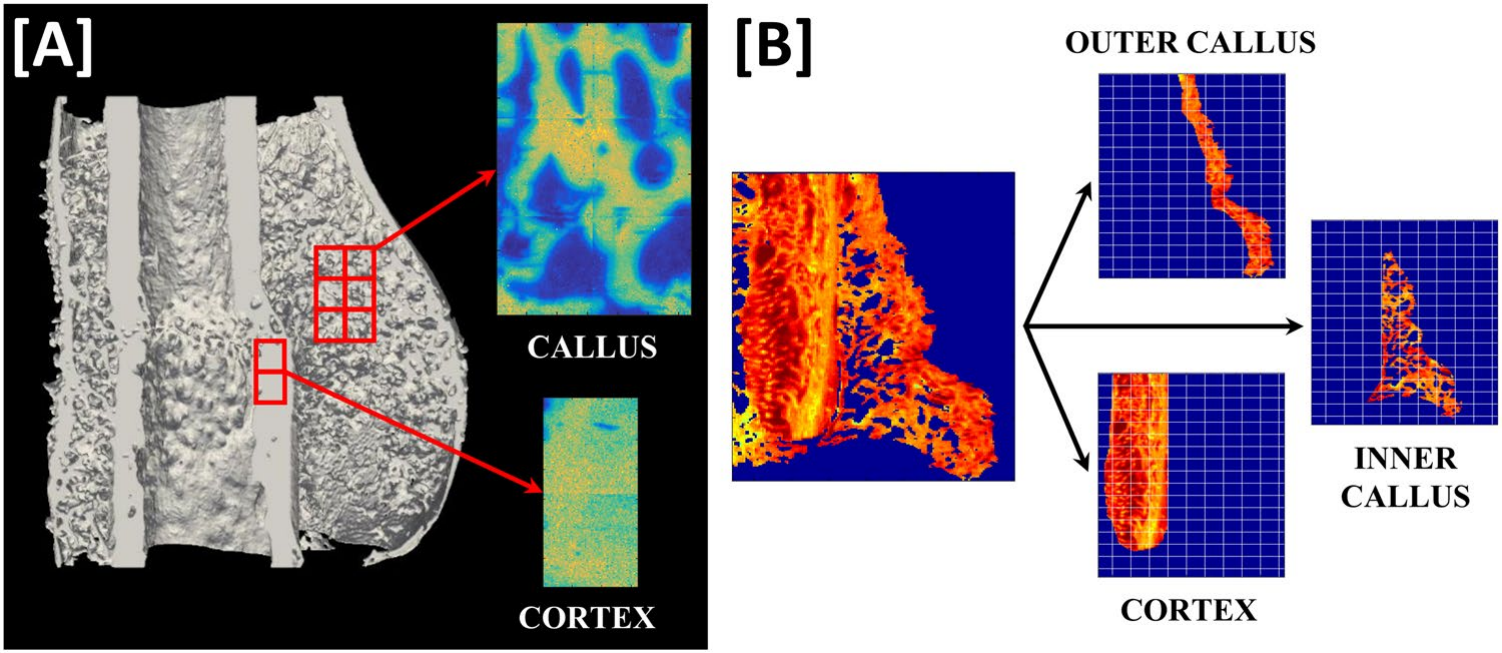
Illustration of the regions of interest for characterization of fracture site composition and nano-structure. (**A**) FTIR spectra were acquired of the depicted inner callus and cortical regions. (**B**) Identical regions in all samples of approximately 5 × 4 mm were scanned with the SAXS/WAXS setups. Measured fracture sites were then sub-divided into inner callus, outer callus and cortical regions of interest as illustrated and parameters for each sample were evaluated within these regions of interest.

Spectra of inner callus and cortical bone at the fracture site were recorded along with reference spectra of epoxy resin and the background window (Fig. 1(A)). Following acquisition, spectra were baseline corrected and IR interference from the epoxy resin subtracted^23,29^. The following four compositional parameters were calculated for each measured region of interest: the mineral/matrix peak area ratio (1200 cm^−1^–900 cm^−1^/1800 cm^−1^–1550 cm^−1^) as an indicator of degree of mineralization^30^, mineral crystallinity (1030 cm^−1^/1020 cm^−1^) representing hydroxyapatite crystal size and perfection^31,32^, collagen maturity (1660 cm^−1^/1690 cm^−1^) based on the ratio of non-reducible (mature) to reducible (immature) cross-links^33^ and acid phosphate content (1127 cm^−1^/1096 cm^−1^) as a measure of newly precipitated crystals^34^. Moreover, the corresponding spatial distribution or heterogeneity of each parameter was calculated based on the full width at half maximum (FWHM) of the pixel histogram of each sample using custom-written scripts in MATLAB (v 9.0 (R2016a), Mathworks Inc.)^23^.

### Scanning Small/Wide angle X-ray Scattering (SAXS/WAXS)

Scanning small/wide angle X-ray scattering (SAXS/WAXS) measurements were conducted at the cSAXS beamline of the Swiss Light Source (PSI Villigen, Switzerland)^35^. Due to constraints in the allocated beamtime at the synchrotron, both SAXS and WAXS measurements were performed on the BMP-7 and the BMP-7 + ZA treated samples (N = 5–7 per group) but only SAXS measurements were conducted on the untreated samples (N = 5–7 per group) (Table 1).

**Table 1:** Overview of the experimental groups, measurement techniques, measurement regions and sample numbers.

		FTIR parameters		SAXS parameters			WAXS parameters		
		Callus	Cortex	Inner Callus	Outer Callus	Cortex	Inner Callus	Outer Callus	Cortex
*Untreated*	*Control*	N = 7	N = 7	N = 7	N = 7	N = 7	—	—	—
	*OVX*	N = 10	N = 10	N = 5	N = 5	N = 5	—	—	—
*BMP*-*7*	*Control*	N = 9	N = 9	N = 7	N = 7	N = 7	N = 6	N = 6	N = 6
	*OVX*	N = 9	N = 9	N = 7	N = 7	N = 7	N = 6	N = 6	N = 6
*BMP-7* + *ZA*	*Control*	N = 8	N = 8	N = 7	N = 7	N = 7	N = 5	N = 5	N = 5
	*OVX*	N = 6	N = 6	N = 7	N = 7	N = 7	N = 5	N = 5	N = 5

**Figure 2:**
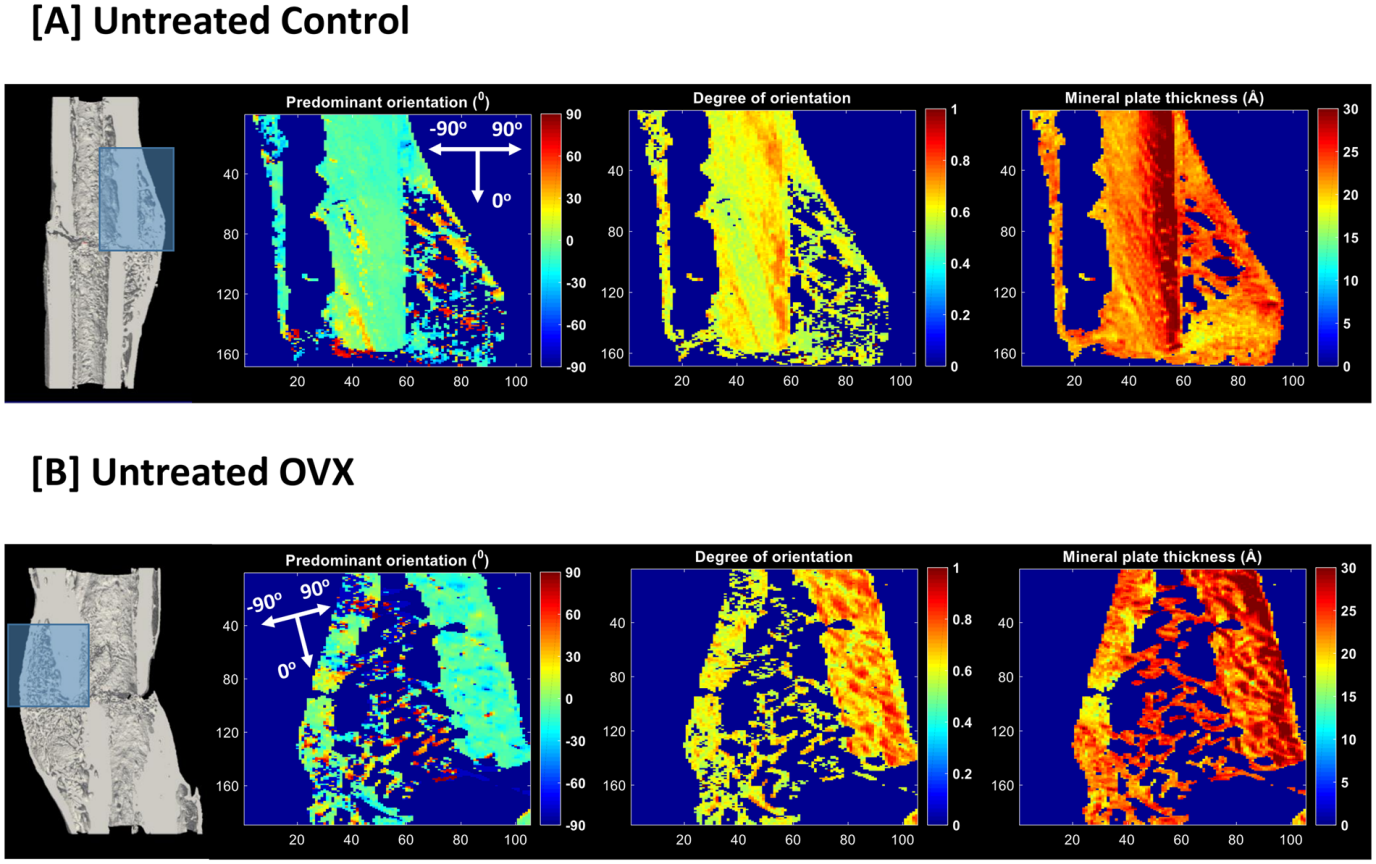
Nanostructural analysis for representative samples in untreated (**A**) Control and (**B**) OVX groups.

Longitudinal, 100 µm thick sections of the embedded femurs were sectioned using a diamond blade saw system under constant water irrigation (EXAKT 300 CP Diamond Band Saw, Norderstedt, Germany). Sections were mounted onto a motorized sample stage with the sample plane perpendicular to the incident beam. Identical areas of approximately 5 x 4 mm^2^ covering the fracture site and callus were scanned with both the SAXS and WAXS setups using a beam size of 20 x 20 μm^2^ (exposure time 50 ms, λ = 1 Å, q-range 0.002–1.7 Å^−1^) (Fig. 2). All 2D scattered intensity images were collected using a PILATUS 2M pixel detector^36^ (PSI Villigen, Switzerland) with sample to detector distance fixed at 7118.5 mm for the SAXS measurements and at 308 mm for the WAXS measurements^19^. Calibration of instrument parameters was achieved by measurement of a Silver-Behenate (AgBH) powder standard.

**Figure 3:**
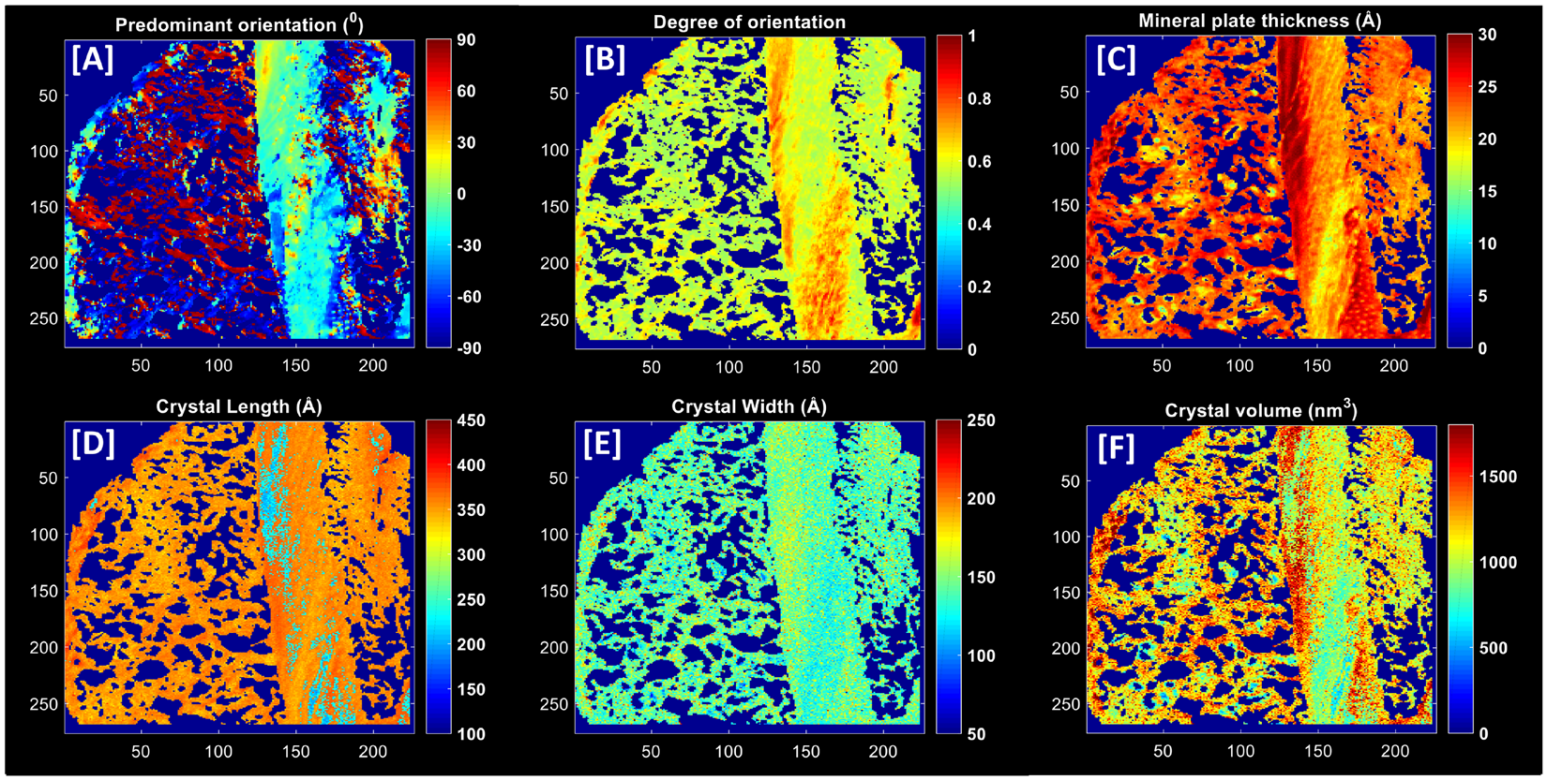
Illustration of the nanostructural analysis for a treated sample with parameter maps of the following parameters: (**A**) Predominant orientation (^0^), (**B**) Degree of orientation, (**C**) Crystal thickness (Å), (**D**) Crystal length (Å), (**E**) Crystal width (Å) and (**F**) Crystal volume (nm^3^).

As detailed below, crystal orientations and dimensions were computed for the scanned SAXS/WAXS regions of the fracture sites (Fig. 3). Measured regions were sub-divided into inner callus, outer callus and cortical regions of interest for the purposes of analysis (Fig. 1(B)).

#### Crystal Orientation Parameters

Analysis of the scattered SAXS intensity spectrum, *I(q, ψ)*, at each measurement point proceeded with radial and azimuthal integration to obtain the *I(q)* and *I(ψ)* intensity distributions respectively, where *q* is the scattering length and *ψ* is the azimuthal angle. Parameters describing crystal orientations were determined by fitting Gaussian curves to the two symmetrical peaks of the azimuthal intensity profile, *I(ψ)*, following the approach detailed in our previous publications^23^. Specifically, the predominant orientation of the crystals (where values range from −90° to 90° denoting the angle of deviation from the long axis of the bone) and the degree of orientation (where 0 denotes randomly orientated crystals and 1 denotes perfectly aligned crystals) were calculated based on the average within each sample volume illuminated by the incident beam (Fig. 3(A,B)).

#### Crystal Dimension Parameters

Mean crystal thickness of the irradiated bone volume was extracted from the azimuthally integrated SAXS intensity distribution *I(q)* by adopting iterative curve fitting to the intensity profile Fig. 3(C))^37^. In brief, the dimensions of the crystals are assumed to be plates that are infinite in two dimensions and of finite thickness, *T*, in the third dimension. In effect, this implies that the two dimensions considered to be infinite in the analysis do not contribute to the scattering range probed in the measurements.

Thus, the expression of the model curve defining the total intensity (for a q-range of 0.32–1.40 nm^−1^) is,

1 $$I(q)=\,C\,{S}_{RPA}(q)\,{P}_{avg}(q)$$

where *P*_*avg*_ represents the average scattering, *S*_*RPA*_ is the random phase approximation (RPA) and *C* is an overall scale factor dependent on sample thickness, mineral content and scattering contrast. A detailed account of its formulation is presented elsewhere^19^.

Iterative weighted non-linear least squares fitting of the model to the *I(q)* curves was performed with the use of automated, custom-written scripts wherein the following 5 parameters were optimized: the crystal thickness *T*, the relative standard deviation of the crystal thickness distribution, a constant background *B*, the scale factor *C* and an RPA parameter as an expression of particle interaction strength. A discussion on the practical aspects of the fitting can be found elsewhere^38^.

Mineral crystal length and crystal width parameters were derived from the radially integrated WAXS intensity profile *I(q)* (Fig. 3(D,E)). The full-widths at half maximum intensity (FWHM) of the (002) and (310) reflections in the diffraction patterns are correlated with mean crystal lengths and widths respectively through the Scherrer equation,

2 $$D=\,\frac{K\lambda }{{\beta }_{1/2}\,\cos \,\theta }$$

where *D* corresponds to the mean crystal dimension contingent on the chosen reflection, *β* represents the FWHM of the peak, *K* is a constant related to crystal shape, *λ* is the wavelength and *θ* is the Bragg angle.

Subsequently, mean crystal volumes were then determined from the product of the respective mean crystal length, width and thickness from each scattered intensity image (Fig. 3(F))^19^. Moreover, measures of the spatial heterogeneity of each nanostructural parameter were determined based on the average standard deviation of all samples within a group.

### Statistical Analysis

Parameters were tested for statistical significance for the following comparisons: (i) healthy vs osteoporotic bone environments, (ii) treatment efficacy or (iii) callus/cortex comparisons, all using a linear mixed effects analysis (R Studio, v1.0, RStudio, Inc.)^39^. The rationale for using a linear mixed effects analysis was to account for the thousands of measurement points per rat instead of reducing these measurement points to a single mean value per rat. In each case, the model construction was as follows. The compositional or nano-structural parameter was defined as the response/dependent variable. Depending on the comparison tested, either the bone environment, treatment group or region was defined as the fixed effect. As random effects, the model accounted for the random contributions of individual rats. Visual inspection of the residuals did not reveal any violations of the assumptions of homoscedasticity and normality. P-values are reported based on likelihood ratio tests with the complete model against a null model omitting the fixed effect. In comparisons of heterogeneity, differences between groups were tested for statistical significance using the Mann-Whitney U test (SPSS, v23, SPSS Inc.). Similarly, statistical differences in heterogeneity parameters between intra-sample callus/cortex regions were determined using the Wilcoxon signed-rank test (SPSS, v23, SPSS Inc.).

### Data Availability

The datasets generated during and/or analysed during the current study are available from the corresponding authors on reasonable request.

## Results

Observed general trends are summarized, and the reader is directed to the Supplementary Material for a complete presentation of results. The Supplementary Material is subdivided into compositional parameters (Supplementary Tables 1–4) and nano-structural parameters (Supplementary Tables 5–7 and Supplementary Figures 1,2). Results include direct comparisons between Control and OVX groups, comparisons between treatment groups within either the Control or the OVX groups, and, finally, comparison between the callus and cortex regions.

Summarized results presented herein are structured under four sections, where the first three sections summarize relevant findings by treatment group: Untreated, BMP-7 only and BMP-7+ ZA. In the final section, relevant callus/cortex comparisons are summarized.

### Untreated: No differences in compositional parameters between untreated Control and OVX fractures but higher degree of crystal orientation in OVX rats

No compositional differences were observed between untreated Control and OVX rats in neither the callus nor the cortex regions (Supplementary Tables 1,2). In Control vs OVX comparisons of inner callus, outer callus and cortical regions, the degree of crystal orientation was significantly increased in the untreated OVX rats compared to untreated Control rats (p < 0.05) (Supplementary Tables 5,6). No other differences in nano-structural parameters were present between untreated Control and OVX fractures (Supplementary Tables 5, 6).

### BMP-7: Increased degree of orientation and greater heterogeneity in crystal dimensions with BMP only in OVX rats compared to Control rats. Greater heterogeneity in crystal dimensions with BMP only compared to other treatment groups

#### Comparisons of corresponding regions in Control vs. OVX groups

Across BMP-only treated inner callus and outer callus regions, a significantly higher degree of crystal orientation was noted in OVX rats relative to corresponding Control rats (p < 0.01) (Supplementary Table 5). In inner callus regions, heterogeneity in crystal thickness, degree of orientation, crystal length and crystal volume were significantly higher in BMP-only treated OVX rats relative to corresponding Controls (p < 0.05, p < 0.01) (Supplementary Table 5). Similarly in outer callus regions, BMP-7 treated OVX rats exhibited greater heterogeneity in crystal volume and degree of orientation relative to Control rats (p < 0.05) (Supplementary Table 5).

#### Greater heterogeneity in crystal dimensions in BMP-only treated rats

**Figure 4:**
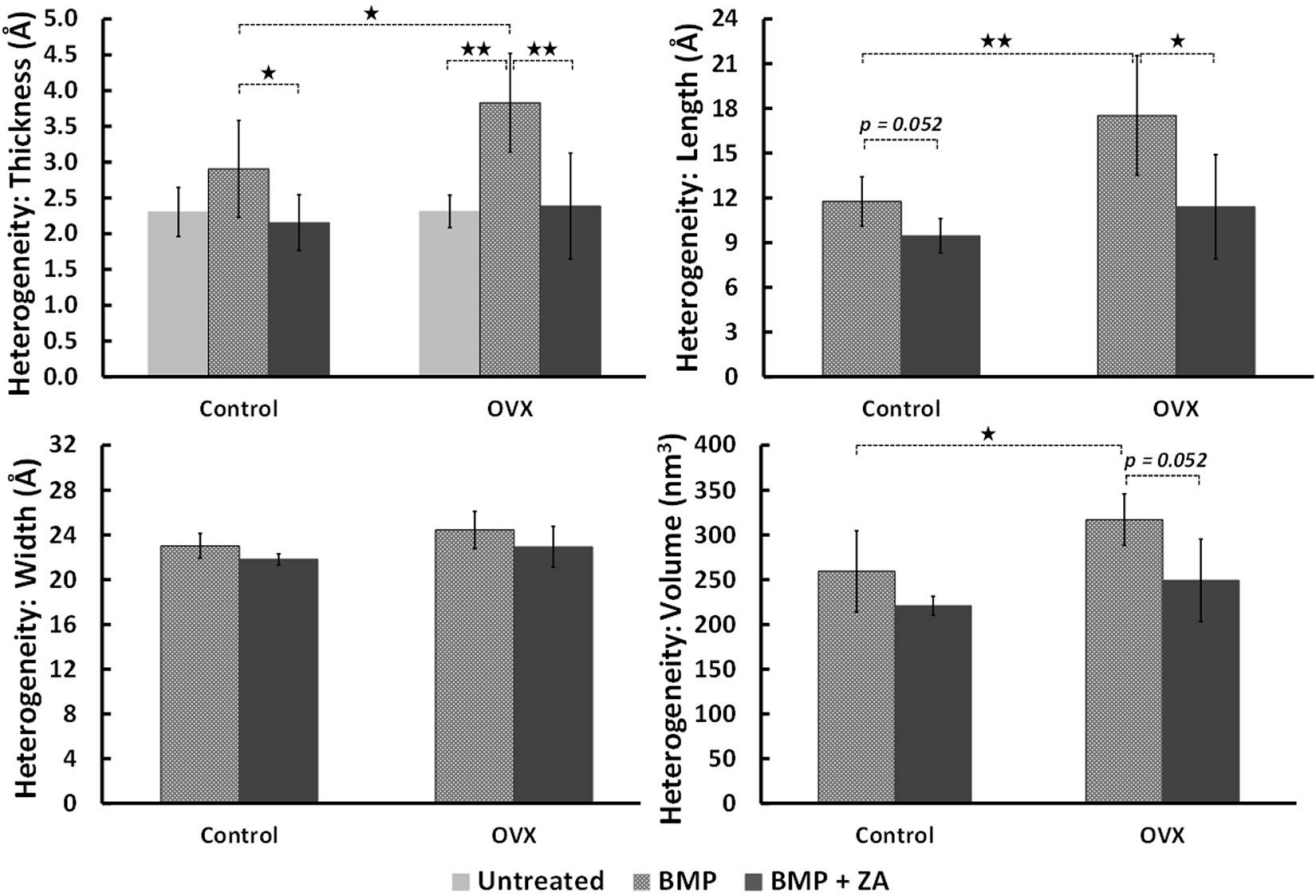
Comparison of heterogeneity in crystal dimensions in the inner callus (*p < 0.05, **p < 0.01).

In Control rats, heterogeneity in crystal thickness of inner callus regions was higher in BMP samples compared to BMP + ZA samples (p < 0.05) and a trend towards higher heterogeneity in crystal length was present with BMP samples compared to BMP + ZA samples (p = 0.052) (Fig. 4; Supplementary Table 7). Comparable trends were present in BMP-only treated osteoporotic rats. Variation in crystal dimensions was in general greater in the BMP-only group compared to the untreated and BMP + ZA groups with higher variation noted in crystal thickness (p < 0.01), crystal length (p < 0.05) and crystal volume (p = 0.052) (Fig. 4; Supplementary Table 7). Both comparisons with p = 0.052 were found to be underpowered by 1–2 samples.

#### Lower degree of orientation and its heterogeneity when treated with BMP-only in both Control and OVX groups compared to no treatment

Degree of orientation was lower in inner callus regions of BMP-only treated groups when compared to untreated groups in both Control and OVX rats (p < 0.05) (Supplementary Table 7). Heterogeneity in degree of orientation was lower in BMP-only groups relative to untreated groups in both Control and OVX rats (p < 0.05, p < 0.01) (Supplementary Table 7).

### BMP-7 + ZA: No differences between Control and OVX rats treated with BMP + ZA. Increased degree of mineralization but lower degree of crystal orientation with BMP + ZA compared to other treatment groups

#### No differences in comparisons of corresponding regions in Control vs. OVX groups

No distinctions in compositional (Supplementary Tables 1,2) and nanostructural (Supplementary Tables 5,6) parameters nor in their heterogeneity was observed between Control and OVX rats treated with BMP + ZA.

#### Increased degree of mineralization in calluses with BMP + ZA treatments compared to no treatment

In both Control and OVX rats, BMP + ZA induced significant increases in the mineral-matrix ratio relative to untreated samples (p < 0.05, p < 0.001) (Supplementary Table 3).

#### Lower degree of orientation and its heterogeneity with BMP + ZA groups relative to untreated or BMP-only treatment groups

In inner callus regions of BMP + ZA treated Control and OVX rats, the degree of orientation and its heterogeneity was lower with BMP + ZA treatment when compared to corresponding BMP-only or no treatment (p < 0.05, p < 0.01, p < 0.001) (Supplementary Table 7).

### Comparison between the inner callus, outer callus and cortex reflects progressive stages of maturity

#### Composition

With regards to the composition, consistently across all groups, the mineral/matrix ratio was lower in the callus than in the cortex (p < 0.001) (Supplementary Table 4). The acid phosphate parameter and its heterogeneity were consistently higher in the callus compared to the cortices across all groups (p < 0.05, p < 0.01, p < 0.001).

#### Crystal Dimensions

Crystal thickness was consistently lower in inner callus regions relative to cortical regions across all groups (p < 0.05, p < 0.001) (Supplementary Figure 1A). In general, heterogeneity in crystal thickness in the inner callus regions of untreated and BMP + ZA treated groups was perceptibly lower than in the cortices (p < 0.05) (Supplementary Figure 2A). However, in the BMP-only treated groups, heterogeneity in crystal thickness in the inner callus regions was greater than or equivalent to that in the cortices (p < 0.05).

Crystal lengths were notably shorter in cortical regions compared to outer callus regions in BMP and BMP + ZA treated groups (p < 0.05) (Supplementary Figure 1B). In general, cortices in these groups exhibited greater heterogeneity in the crystal length relative to inner and outer callus regions (p < 0.05) (Supplementary Figure 2B).

Crystal width in BMP-only treatment groups was widest in the outer callus (p < 0.05, p < 0.01, p < 0.001) (Supplementary Figure 1C). Heterogeneity in crystal widths was greater in inner callus regions of all BMP and BMP + ZA treated groups relative to cortices (p < 0.05) (Supplementary Figure 2C).

#### Crystal Orientations

Predominant orientation in inner callus regions of untreated groups differed significantly from the cortex (p < 0.05, p < 0.001) (Supplementary Figure 1E). Furthermore, in untreated control fractures, predominant orientation in the three regions were distinct with the inner callus furthest from 0°, followed by the outer callus with the mean predominant orientation of the cortex closest to 0° (p < 0.05, p < 0.01, p < 0.001) (Supplementary Figure 1E). However, in untreated OVX fractures, a clear distinction was only present between inner callus and cortex. Consistently across all groups, heterogeneity in predominant orientation was significantly greater in inner callus and outer callus regions compared to cortices (p < 0.05) (Supplementary Figure 2E).

Differences in the degree of orientation were consistent across all treatment groups. The degree of orientation was greater in cortices compared to inner and outer callus regions (p < 0.05, p < 0.01, p < 0.001) (Supplementary Figure 1F). Heterogeneity in the degree of orientation was significantly greater in the inner callus of untreated control fractures than the cortex (p < 0.05). In the presence of BMP or BMP + ZA, this relationship reversed with a greater heterogeneity in the degree of orientation of the cortex than the inner callus (p < 0.05) (Supplementary Figure 2F). These observations were less clearly discernible in corresponding OVX groups.

## Discussion

The objective of the present study was to investigate the compositional and nano-structural basis of fracture healing in healthy and osteoporotic bone. Literature on healing of bones in osteoporotic patients and animal models are inconclusive with contradictory findings reported on the hypothesis that an estrogen-deficient environment constitutes a greater challenge for skeletal regeneration^6^. Our results show that on the basis of compositional and nano-structural characterizations, the osseous healing response after six weeks in healthy and osteoporotic rat femoral fractures is fundamentally the same. Furthermore, the influence of BMP-7 mediated osteogenic stimulation in an estrogen-deficient environment is distinguished by greater heterogeneity in the nanostructural crystal dimensions. Finally, the capacity of ZA to mitigate the upregulated catabolic effects of BMP-7 and an osteoporotic bone environment is underscored.

**Figure 5:**
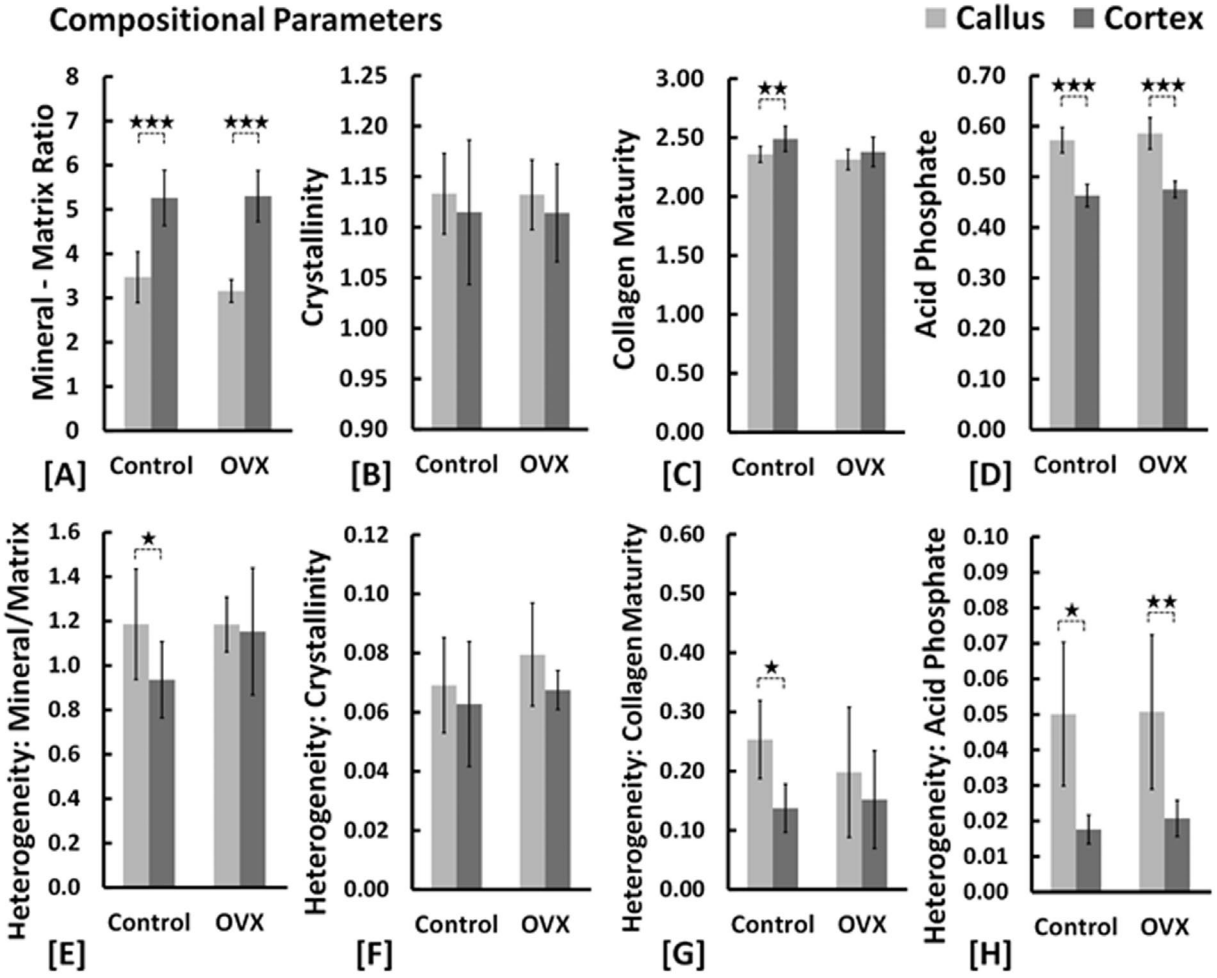
Comparisons of compositional parameters in healthy and osteoporotic fractures (*p < 0.05, **p < 0.01).

**Figure 6:**
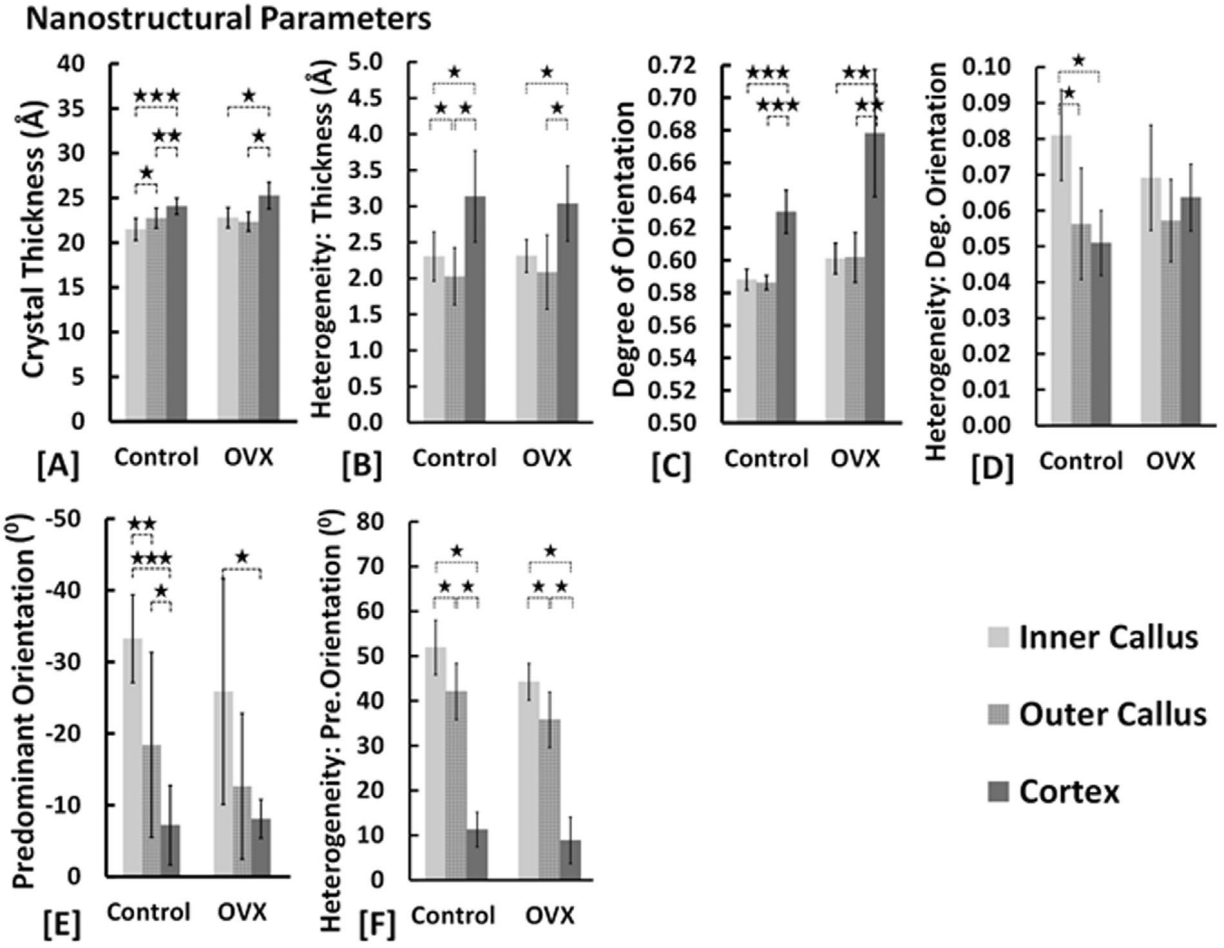
Comparisons of nanostructural parameters in healthy and osteoporotic fractures (*p < 0.05).

It can be proposed that the inner callus, the outer callus and cortices represent regions of progressively increasing degrees of bone maturity. Our compositional and nano-structural data is supportive of this understanding. Compositionally, the immaturity of the fracture callus is in line with expectations reflecting a lower degree of mineralization and collagen maturity and increased acid phosphate deposition relative to cortices (Fig. 5)^20,23^. This coincides with greater spatial heterogeneity in the mineral-matrix ratio, collagen maturity and acid phosphate substitution (Fig. 5). In agreement with previously reported values, no distinction between callus and cortex was present in comparisons of the crystallinity parameter (Fig. 5(B))^20^. Nano-structurally, the characterization of the inner callus, the outer callus and cortex as progressively increasing degrees of bone maturity holds firm. With respect to each successive region, mineral crystal thickness increased while the deviation of the predominant orientation from the longitudinal axis decreased (Fig. 6). Callus regions exhibit a lower degree of orientation compared to cortices (Fig. 6). The heterogeneity in the predominant orientation reflects the progressive degrees of maturity with the least variation in the cortex, followed by the outer callus region and highest in the inner callus region (Fig. 6(F)). In a similar sense, the heterogeneity in the degree of orientation is markedly higher in the inner callus compared to the outer callus and cortical regions (Fig. 6(D)).

Interestingly, heterogeneity in the crystal thickness parameter deviated from this model and exhibited the greatest variation in the cortex, followed by the inner callus and then the outer callus regions (Fig. 6(B)). It can be inferred that in the cortex this is a consequence of the mature intracortical regions combined with the active new bone formation on the periosteal and endosteal surfaces^19^. Similarly, the surfaces of the trabecular-like structures of the inner callus are sites of high activity but variation is significantly lower than cortical regions as it lacks the mature inner cortical regions. The dense, lamellar-structured outer callus exhibits less surface area and relatively mature bone, thus the lowest variation is observed here. Conversely, heterogeneity in the orientation parameters of the inner callus are influenced by the direction in which individual trabeculae grow and are therefore markedly much higher than the outer callus and cortical regions. Thus, there appears to be a distinction between heterogeneity in crystal orientation parameters and heterogeneity in crystal thickness.

Comparisons with reported differences between the callus and the cortex in the literature are challenging as previous publications are based on different animal models and generally have very low sample sizes^23,25,26^. Liu *et al*. reported SAXS parameters in an osteotomized sheep model with a single sample at weeks 2, 3, 6 and 9 following surgery^25^. Periosteal callus regions exhibited lower degrees of orientation and crystal thicknesses than the cortex at all time points with the exception of week 9 where mean crystal thickness values in the callus exceeded those of the cortex. Similarly, Hoerth *et al*. investigated nano-structural changes in a rat osteotomy model of non-critical (1 mm gap) and critical (5 mm gap) fracture healing at weeks 2, 3, 4 and 6 post-surgery^26^. Degree of orientation exhibited a similar trend to the findings of Liu *et al*. with progressive increases observed with values reaching parity with the cortex at 6 weeks. At all time points, crystal thickness in callus regions were equivalent or higher than cortical regions, with gradients outwards from the osteotomy site exhibiting a gradual increase with maximal values in the outer periosteal callus. The authors concluded that crystal dimensions morphed following deposition with growth from short and thick crystals to long and thin crystals. Our results are not fully supportive of this conclusion. Foremost, at six weeks, crystal thicknesses in callus regions were significantly lower than the cortex (Fig. 5). Furthermore, the crystal lengths in our treated samples were longer in callus regions than the cortex (Supplementary Figure 1B). However, all of the aforementioned studies in the literature share a similar approach in their calculation of the mineral crystal thickness parameter based on an assumption that the mineral phase volume fraction is 0.5 for bone^40,41^. This is questionable in the context of newly formed bone and potentially leads to over-estimations of the mineral crystal thickness. The curve-fitting approach used in this manuscript is independent of any assumption on mineral volume fraction and an assessment of its merits has been previously published^19,27,38^.

Does osteoporosis alter this paradigm? Across all structural and compositional parameters, the degree of orientation was the only parameter in which a significant difference was observed in direct comparisons of healthy and osteoporotic fracture site regions. Callus regions and cortices of osteoporotic samples exhibited a significantly higher ordering of the crystals relative to corresponding regions in healthy samples (Supplementary Tables 5,6). Ovariectomy-induced osteoporosis is characterized by a higher remodelling rate with an imbalance that favours excessive resorption. Interpreting the degree of orientation parameter as a marker of maturity suggests that this augmented resorptive effect is primarily concentrated on newly-formed, younger bone. Notably, the effect is more pronounced in the OVX cortex than the OVX inner callus reflecting the relative ages of the respective bone regions with the cortex exposed to 18 weeks of estrogen-deficiency compared to only 6 weeks for the inner callus.

In healthy samples, distinctions in the relative maturities of the inner callus, outer callus and cortical regions or distinctions between callus and cortices were present. In OVX samples, these relative differences are retained but are less distinct in that they do not all achieve statistical significance. Figures 5 and 6 are illustrative of this. Nevertheless, cumulatively the data suggests that compositionally and nano-structurally fracture healing is essentially unaltered in an osteoporotic bone environment. Indeed, the femurs in this study were also subject to radiological, micro-structural and mechanical measures of bone quality and the findings (reported in a separate publication) are also in agreement with our conclusion^6^. However, the relative lack of significant differences in the nano-structural characterization merits a note of caution as it is conceivable that the sample sizes of N = 5–7 may have been underpowered to detect significant differences. To test this, a representative power analysis was performed where a significant difference was expected but not found (power = 0.8, α = 0.05). In this instance, the sample size was determined to be N = 9 to detect a significant difference. Thus, our rationale in using a linear mixed effects analysis is to account for the thousands of measurement points per rat instead of a single mean value per rat.

Does the osseous healing response differ between healthy and osteoporotic bone environments when subjected to treatments? In our radiological, micro-structural and mechanical evaluations of BMP-7 treated healthy and OVX fractures, an increased sensitivity to BMP-7 was observed in osteoporotic rats^6^. Nano-structurally, there is some evidence to support this conclusion. Namely, an augmented heterogeneity in crystal dimensions is observed in the inner callus of BMP-7 only treated healthy and osteoporotic fractures. In control fractures, heterogeneity in crystal thickness and length were higher in BMP-7 only samples compared to BMP-7 + ZA samples (Fig. 4). Similarly, in osteoporotic fractures, heterogeneity in crystal thickness, length and volume were higher in the BMP-7 only group compared to untreated and/or BMP + ZA groups (Fig. 4). It should be noted that no WAXS data was available for the untreated samples and thus no comparisons are possible for the crystal length, width and volume parameters. Moreover, there were additional distinctions between healthy and osteoporotic samples treated with BMP-7. Specifically, heterogeneity in crystal thickness, length, volume and degree of orientation were higher in BMP-only treated osteoporotic samples relative to BMP-only treated controls (Fig. 4).

Greater heterogeneity in crystal dimensions can be attributed to the BMP-induced stimulation of both anabolic and catabolic processes during fracture repair. Furthermore, it can be postulated that greater heterogeneity in crystal dimensions is thought to augment fracture resistance based on the understanding that heterogeneous material properties are intrinsic toughening mechanisms in bone^42,43^. The basis of this hypothesis is supported by observations in older or osteoporotic animal bone of a shift in crystal size distribution characterized by a surplus of larger crystals. Vennin *et al*. also found significantly greater variance in nano-indentation parameters of biopsies from post-menopausal women with no fracture history compared to age/BMD-matched controls with osteoporotic fractures^44^. Moreover, Tai *et al*. characterized nano-mechanical properties of bone and used computational simulations to attribute nano-scale structural heterogeneity to enhanced ductility and energy dissipation^16^. Therefore, it could be inferred that healthy bone is optimally distinguished by a broad distribution of crystal sizes^42,43^. Nevertheless, caution is advised in interpreting this finding as further evidence is needed to substantiate the link between crystal heterogeneity and macro-scale mechanical properties.

Finally, in our previous publication, we concluded that adoption of an anabolic–anti-catabolic approach is better suited for pharmacological interventions on the basis of radiological, microstructural and mechanical data^6^. The compositional and nano-structural data presented in this study supports that conclusion. In both healthy and osteoporotic bone environments, BMP + ZA significantly increased the mineral – matrix ratio when compared to untreated femurs (Supplementary Table 3). In direct comparisons between Control and OVX groups, OVX rats exhibited a significantly higher degree of orientation in callus regions of both untreated and BMP-only treated groups (Supplementary Table 5). This was not observed with BMP + ZA. Thus, as expected, ZA mitigated the upregulated catabolic effects of both BMP-7 and an osteoporotic bone environment. In comparisons between treatment groups, the degree of orientation was lower in BMP + ZA groups relative to untreated or BMP-only treatment groups (Supplementary Table 7). This is in agreement with our previously reported histological observations where immature bone predominated within the substantially enlarged BMP + ZA callus^12^. Furthermore, the increases in crystal heterogeneity observed with BMP are not present with BMP + ZA as the net result is a strong anabolic effect.

## Electronic supplementary material

Supplementary Information
