# Supplementary material for: The compositional and nano-structural basis of fracture healing in healthy and osteoporotic bone
Source: Sci Rep. 2018 Jan 25;8:1591. doi: 10.1038/s41598-018-19296-z (PMC5785543; doi:10.1038/s41598-018-19296-z)
Supplement: Supplementary file 1 — Supplementary Information [file 41598_2018_19296_MOESM1_ESM.pdf]

Supplementary Material

The compositional and nano-structural basis of fracture healing in healthy and osteoporotic bone.

Neashan Mathavan, Mikael J. Turunen, Manuel Guizar-Sicairos, Martin Bech, Florian Schaff, Magnus Tägil, Hanna Isaksson

Supplementary Material: Composition

Supplementary Table 1. Callus : Control vs. OVX comparisons.
Supplementary Table 2. Cortex : Control vs. OVX comparisons.
Supplementary Table 3. Treatment efficacy.
Supplementary Table 4. Callus/Cortex comparisons.

Supplementary Material: Nanostructure

Supplementary Table 5. Callus : Control vs. OVX comparisons.
Supplementary Table 6. Cortex : Control vs. OVX comparisons.
Supplementary Table 7. Treatment efficacy.
Supplementary Figure 1. Callus/Cortex comparisons.
Supplementary Figure 2. Callus/Cortex comparisons: Heterogeneity.

Supplementary Table 1. Comparisons between healthy and osteoporotic fracture repair of callus compositional parameters (** $p < 0.01$).

			Callus (Compositional Parameters)											
			Mineral - Matrix Ratio			Crystallinity 1030 / 1020			Collagen Maturity 1660 / 1690			Acid Phosphate 1127 / 1096		
Treatment		N	Mean	SD	p-value	Mean	SD	p-value	Mean	SD	p-value	Mean	SD	p-value
Untreated	Control	7	3.47	0.57		1.13	0.04		2.36	0.07		0.57	0.03	
	OVX	10	3.16	0.26		1.13	0.03		2.31	0.09		0.59	0.03	
BMP	Control	9	3.66	0.57		1.10	0.04		2.40	0.09	**	0.58	0.03	
	OVX	9	3.57	0.58		1.11	0.03		2.27	0.10		0,58 (^)	0.02	
BMP + ZA	Control	8	4.18	0.68		1.10	0.05		2.34	0.10		0.59	0.04	
	OVX	6	3.98	0.36		1.12	0.04		2.32	0.06		0.57	0.02	

			Callus (Compositional Parameters - Heterogeneity)											
			Mineral - Matrix Ratio Heterogeneity			Crystallinity Heterogeneity			Collagen Maturity Heterogeneity			Acid Phosphate Heterogeneity		
Treatment		N	Mean	SD	p-value	Mean	SD	p-value	Mean	SD	p-value	Mean	SD	p-value
Untreated	Control	7	1,19 (#)	0.25		0.07	0.02		0.25	0.07		0.05	0.02	
	OVX	10	1.18	0.12		0.08	0.02		0,20 (§)	0.11		0.05	0.02	
BMP	Control	9	0.98	0.20		0.07	0.01		0.27	0.17		0.05	0.04	
	OVX	9	1.09	0.22		0.07	0.02		0.29	0.16		0.05	0.01	
BMP + ZA	Control	8	1.11	0.16		0.06	0.01		0.28	0.11		0.05	0.02	
	OVX	6	1.15	0.18		0.06	0.02		0.34	0.19		0,04 (Ω)	0.01	

(Ω) denotes N = 5
(#) denotes N = 6
(^) denotes N = 8
(§) denotes N = 9

Supplementary Table 2. Comparisons between healthy and osteoporotic fracture repair of cortical compositional parameters.

			Cortex (Compositional Parameters)											
			**Mineral - Matrix Ratio**			**Crystallinity 1030 / 1020**			**Collagen Maturity 1660 / 1690**			**Acid Phosphate 1127 / 1096**		
**Treatment**		**N**	Mean	SD	p-value	Mean	SD	p-value	Mean	SD	p-value	Mean	SD	p-value
Untreated	Control	7	5.26	0.63		1.11	0.07		2.49	0.11		0.46	0.02	
	OVX	10	5.30	0.58		1.11	0.05		2.38	0.12		0.48	0.02	
BMP	Control	9	5.75	0.78		1.07	0.05		2.47	0.09		0.47	0.04	
	OVX	9	5.71	0.53		1.09	0.06		2.46	0.13		0,47 (#)	0.01	
BMP + ZA	Control	8	5.47	0.72		1.09	0.07		2.49	0.12		0.48	0.02	
	OVX	6	5.97	0.74		1.12	0.07		2.45	0.12		0.48	0.00	

			Cortex (Compositional Parameters - Heterogeneity)											
			**Mineral - Matrix Ratio Heterogeneity**			**Crystallinity Heterogeneity**			**Collagen Maturity Heterogeneity**			**Acid Phosphate Heterogeneity**		
**Treatment**		**N**	Mean	SD	p-value	Mean	SD	p-value	Mean	SD	p-value	Mean	SD	p-value
Untreated	Control	7	0.94	0.17		0.06	0.02		0.14	0.04		0.02	0.00	
	OVX	10	1.15	0.29		0.07	0.01		0,15 (#)	0.08		0.02	0.01	
BMP	Control	9	0.95	0.15		0.06	0.01		0.16	0.07		0.02	0.00	
	OVX	9	0.91	0.18		0.06	0.01		0.14	0.08		0.02	0.01	
BMP + ZA	Control	8	0.86	0.17		0.06	0.01		0.17	0.11		0.02	0.02	
	OVX	6	1.00	0.15		0.06	0.02		0.17	0.10		0.02	0.01	

(#) denotes N = 8

Supplementary Table 3. Comparison of Treatments: Comparisons of inner callus regions are presented between untreated, BMP-7 and BMP-7 + ZA treatments within either the healthy or the osteoporotic groups.
(***** $p < 0.05$, ****** $p < 0.01$, ******* $p < 0.001$ when compared to Untreated femurs, and
o $p < 0.05$, **oo** $p < 0.01$, **ooo** $p < 0.001$ when compared to BMP only treated femurs).

			Control (Compositional Parameters)											
			Mineral - Matrix Ratio			Crystallinity 1030 / 1020			Collagen Maturity 1660 / 1690			Acid Phosphate 1127 / 1096		
**Treatment**		**N**	Mean	SD	p-value	Mean	SD	p-value	Mean	SD	p-value	Mean	SD	p-value
Untreated	Control	7	3.47	0.57		1.13	0.04		2.36	0.07		0.57	0.03	
BMP	Control	9	3.66	0.57		1.10	0.04		2.40	0.09		0.58	0.03	
BMP + ZA	Control	8	4.18	0.68	*	1.10	0.05		2.34	0.10		0.59	0.04	

			OVX (Compositional Parameters)											
			Mineral - Matrix Ratio			Crystallinity 1030 / 1020			Collagen Maturity 1660 / 1690			Acid Phosphate 1127 / 1096		
**Treatment**		**N**	Mean	SD	p-value	Mean	SD	p-value	Mean	SD	p-value	Mean	SD	p-value
Untreated	OVX	10	3.16	0.26		1.13	0.03		2.31	0.09		0.59	0.03	
BMP	OVX	9	3.57	0.58		1.11	0.03		2.27	0.10		0,58	0.02	
BMP + ZA	OVX	6	3.98	0.36	***	1.12	0.04		2.32	0.06		0.57	0.02	

			Control (Compositional Parameters - Heterogeneity)											
			Mineral - Matrix Ratio Heterogeneity			Crystallinity Heterogeneity			Collagen Maturity Heterogeneity			Acid Phosphate Heterogeneity		
**Treatment**		**N**	Mean	SD	p-value	Mean	SD	p-value	Mean	SD	p-value	Mean	SD	p-value
Untreated	Control	7	1,19 (#)	0.25		0.07	0.02		0.25	0.07		0.05	0.02	
BMP	Control	9	0.98	0.20		0.07	0.01		0.27	0.17		0.05	0.04	
BMP + ZA	Control	8	1.11	0.16		0.06	0.01		0.28	0.11		0.05	0.02	

(#) denotes N = 6

			OVX (Compositional Parameters - Heterogeneity)											
			Mineral - Matrix Ratio Heterogeneity			Crystallinity Heterogeneity			Collagen Maturity Heterogeneity			Acid Phosphate Heterogeneity		
**Treatment**		**N**	Mean	SD	p-value	Mean	SD	p-value	Mean	SD	p-value	Mean	SD	p-value
Untreated	OVX	10	1.18	0.12		0.08	0.02		0,20 (§)	0.11		0.05	0.02	
BMP	OVX	9	1.09	0.22		0.07	0.02		0.29	0.16		0.05	0.01	
BMP + ZA	OVX	6	1.15	0.18		0.06	0.02		0.34	0.19	*	0,04 (Ω)	0.01	

(Ω) denotes N = 5
(§) denotes N = 9

Supplementary Table 4. Comparisons between callus and cortex regions. (* p < 0.05, ** p < 0.01, *** p < 0.001).

		Mineral-Matrix					Mineral-Matrix (Heterogeneity)				
		CALLUS		CORTEX			CALLUS		CORTEX		
	N	*Mean*	*SD*	*Mean*	*SD*	*p-value*	*Mean*	*SD*	*Mean*	*SD*	*p-value*
Untreated (Control)	7	3.47	0.57	5.26	0.63	***	1.19	0.25	0.94	0.17	*
Untreated (OVX)	10	3.16	0.26	5.30	0.58	***	1.18	0.12	1.15	0.29	
BMP (Control)	9	3.66	0.57	5.75	0.78	***	0.98	0.20	0.95	0.15	
BMP (OVX)	9	3.57	0.58	5.71	0.53	***	1.09	0.22	0.91	0.18	
BMP + ZA (Control)	8	4.18	0.68	5.47	0.72	***	1.11	0.16	0.86	0.17	*
BMP + ZA (OVX)	6	3.98	0.36	5.97	0.74	***	1.15	0.18	1.00	0.15	*

		Crystallinity					Crystallinity (Heterogeneity)				
		CALLUS		CORTEX			CALLUS		CORTEX		
	N	*Mean*	*SD*	*Mean*	*SD*	*p-value*	*Mean*	*SD*	*Mean*	*SD*	*p-value*
Untreated (Control)	7	1.13	0.04	1.11	0.07		0.07	0.02	0.06	0.02	
Untreated (OVX)	10	1.13	0.03	1.11	0.05		0.08	0.02	0.07	0.01	
BMP (Control)	9	1.10	0.04	1.07	0.05	*	0.07	0.01	0.06	0.01	*
BMP (OVX)	9	1.11	0.03	1.09	0.06		0.07	0.02	0.06	0.01	
BMP + ZA (Control)	8	1.10	0.05	1.09	0.07		0.06	0.01	0.06	0.01	
BMP + ZA (OVX)	6	1.12	0.04	1.12	0.07		0.06	0.02	0.06	0.02	

		Collagen Maturity					Collagen Maturity (Heterogeneity)				
		CALLUS		CORTEX			CALLUS		CORTEX		
	N	*Mean*	*SD*	*Mean*	*SD*	*p-value*	*Mean*	*SD*	*Mean*	*SD*	*p-value*
Untreated (Control)	7	2.36	0.07	2.49	0.11	**	0.25	0.07	0.14	0.04	*
Untreated (OVX)	10	2.31	0.09	2.38	0.12		0.20	0.11	0.15	0.08	
BMP (Control)	9	2.40	0.09	2.47	0.09	*	0.27	0.17	0.16	0.07	
BMP (OVX)	9	2.27	0.10	2.46	0.13	**	0.29	0.16	0.14	0.08	*
BMP + ZA (Control)	8	2.34	0.10	2.49	0.12	**	0.28	0.11	0.17	0.11	
BMP + ZA (OVX)	6	2.32	0.06	2.45	0.12	*	0.34	0.19	0.17	0.10	

		Acid Phosphate					Acid Phosphate (Heterogeneity)				
		CALLUS		CORTEX			CALLUS		CORTEX		
	N	*Mean*	*SD*	*Mean*	*SD*	*p-value*	*Mean*	*SD*	*Mean*	*SD*	*p-value*
Untreated (Control)	7	0.573	0.025	0.463	0.022	***	0.050	0.020	0.018	0.004	*
Untreated (OVX)	10	0.586	0.031	0.475	0.016	***	0.051	0.022	0.021	0.005	**
BMP (Control)	9	0.584	0.029	0.467	0.040	***	0.052	0.040	0.021	0.004	*
BMP (OVX)	9	0.585	0.025	0.468	0.013	***	0.052	0.013	0.023	0.014	*
BMP + ZA (Control)	8	0.587	0.042	0.476	0.023	***	0.048	0.025	0.025	0.018	*
BMP + ZA (OVX)	6	0.573	0.024	0.478	0.002	***	0.040	0.015	0.025	0.009	*

Supplementary Table 5. Comparisons between healthy and osteoporotic fracture repair of nanostructural parameters in callus regions (* $p < 0.05$, ** $p < 0.01$).

			Inner Callus (Nanostructural parameters)																	
			Crystal Thickness (Å)			Predominant Orientation (degrees)			Degree of Orientation			D 002 - Crystal Length (nm)			D 310 - Crystal Width (nm)			Crystal Volume (nm^3)		
**Treatment**		**N**	Mean	SD	p-value	Mean	SD	p-value	Mean	SD	p-value	Mean	SD	p-value	Mean	SD	p-value	Mean	SD	p-value
Untreated	Control	7	21.5	1.23		-33.2	6.14		0.59	0.006	*	-	-	-	-	-	-	-	-	-
	OVX	5	22.8	1.14		-25.9	15.7		0.60	0.009		-	-	-	-	-	-	-	-	-
BMP	Control	6 - 7	22.3	0.92		-2.7	17.1		0.58	0.009	**	346.9	2.05		139.2	2.04		1093	62	
	OVX	6 - 7	22.6	1.85		-14.1	28.6		0.59	0.005		350.0	3.87		141.3	3.69		1141	134	
BMP + ZA	Control	5 - 7	21.8	0.89		-13.4	26.9		0.57	0.008		346.3	2.67		140.7	2.12		1077	49	
	OVX	5 - 7	22.4	1.22		0.48	24.9		0.58	0.015		349.43	4.74		140.7	4.61		1100	111	

			Inner Callus (Nanostructural parameters - Heterogeneity)																	
			Crystal Thickness (Å)			Predominant Orientation (degrees)			Degree of Orientation			D 002 - Crystal Length (nm)			D 310 - Crystal Width (nm)			Crystal Volume (nm^3)		
**Treatment**		**N**	Mean	SD	p-value	Mean	SD	p-value	Mean	SD	p-value	Mean	SD	p-value	Mean	SD	p-value	Mean	SD	p-value
Untreated	Control	7	2.30	0.34		51.9	6.09		0.081	0.01		-	-	-	-	-	-	-	-	-
	OVX	5	2.31	0.23		44.3	4.09		0.069	0.01		-	-	-	-	-	-	-	-	-
BMP	Control	6 - 7	2.90	0.68	*	66.0	9.13		0.045	0.006	*	11.8	1.65	**	23.0	1.11		259	45	*
	OVX	6 - 7	3.83	0.69		54.7	13.6		0.055	0.007		17.5	4.00		24.5	1.66		317	29	
BMP + ZA	Control	5 - 7	2.16	0.39		57.4	14.8		0.038	0.004		9.5	1.15		21.8	0.50		221	11	
	OVX	5 - 7	2.39	0.74		64.65	7.40		0.043	0.006		11.40	3.49		23.0	1.83		249	46	

			Outer Callus (Nanostructural parameters)																	
			Crystal Thickness (Å)			Predominant Orientation (degrees)			Degree of Orientation			D 002 - Crystal Length (Å)			D 310 - Crystal Width (Å)			Crystal Volume (nm^3)		
**Treatment**		**N**	Mean	SD	p-value	Mean	SD	p-value	Mean	SD	p-value	Mean	SD	p-value	Mean	SD	p-value	Mean	SD	p-value
Untreated	Control	7	22.8	1.13		-18.41	12.91		0.59	0.004	*	-	-	-	-	-	-	-	-	-
	OVX	5	22.4	1.07		-12.64	10.18		0.60	0.015		-	-	-	-	-	-	-	-	-
BMP	Control	6 - 7	23.0	0.72		3.0	12.3		0.58	0.01	**	348.9	3.5		144.3	2.62	*	1166	59	
	OVX	6 - 7	24.7	2.60		-4.50	19.04		0.61	0.03		354.2	10.8		147.9	2.64		1308	193	
BMP + ZA	Control	5 - 7	22.9	1.02		-0.6	9.9		0.58	0.01		350.3	2.7		144.7	0.95		1181	44	
	OVX	5 - 7	23.8	1.27		1.0	22.1		0.59	0.02		353.5	3.5		146.0	3.56		1256	109	

			Outer Callus (Nanostructural parameters - Heterogeneity)																	
			Crystal Thickness (Å)			Predominant Orientation (degrees)			Degree of Orientation			D 002 - Crystal Length (nm)			D 310 - Crystal Width (nm)			Crystal Volume (nm^3)		
**Treatment**		**N**	Mean	SD	p-value	Mean	SD	p-value	Mean	SD	p-value	Mean	SD	p-value	Mean	SD	p-value	Mean	SD	p-value
Untreated	Control	7	2.03	0.40		42.1	6.28		0.056	0.02		-	-	-	-	-	-	-	-	-
	OVX	5	2.09	0.51		35.77	6.20		0.057	0.01		-	-	-	-	-	-	-	-	-
BMP	Control	6 - 7	2.02	0.10		56.7	10.06	*	0.042	0.004	*	18.5	9.0		18.8	0.33		213	8	*
	OVX	6 - 7	2.88	1.12		41.1	15.78		0.058	0.013		24.5	15.7		20.6	1.78		302	69	
BMP + ZA	Control	5 - 7	2.03	0.32		49.0	16.36		0.043	0.005		13.4	2.1		20.3	2.34		226	25	
	OVX	5 - 7	2.36	0.66		53.6	7.60		0.052	0.014		15.5	2.4		20.8	1.24		261	31	

Supplementary Table 6. Comparisons between healthy and osteoporotic fracture repair of nanostructural parameters in cortical regions (***** p < 0.05 , ****** p < 0.01).

			Cortex (Nanostructural parameters)																	
			Crystal Thickness (Å)			Predominant Orientation (degrees)			Degree of Orientation			D 002 - Crystal Length (Å)			D 310 - Crystal Width (Å)			Crystal Volume (nm^3)		
**Treatment**		**N**	Mean	SD	p-value	Mean	SD	p-value	Mean	SD	p-value	Mean	SD	p-value	Mean	SD	p-value	Mean	SD	p-value
Untreated	Control	7	24.1	0.90		-7.20	5.55		0.63	0.01	*	-	-	-	-	-	-	-	-	-
	OVX	5	25.3	1.49		-8.08	2.70		0.68	0.04		-	-	-	-	-	-	-	-	-
BMP	Control	6 - 7	25.0	2.11		-4.8	5.1		0.65	0.02		303.4	39.9		141.5	2.12		1075	188	
	OVX	6 - 7	25.4	2.92		-3.36	8.80		0.68	0.03		316.4	49.3		141.2	1.23		1130	278	
BMP + ZA	Control	5 - 7	25.7	1.90		-3.2	4.6		0.66	0.03		336.6	14.8		143.7	3.78		1264	150	
	OVX	5 - 7	24.6	1.12		-5.8	8.3		0.65	0.01		328.1	26.4		142.6	0.88		1167	111	

			Cortex (Nanostructural parameters - Heterogeneity)																	
			Crystal Thickness (Å)			Predominant Orientation (degrees)			Degree of Orientation			D 002 - Crystal Length (nm)			D 310 - Crystal Width (nm)			Crystal Volume (nm^3)		
**Treatment**		**N**	Mean	SD	p-value	Mean	SD	p-value	Mean	SD	p-value	Mean	SD	p-value	Mean	SD	p-value	Mean	SD	p-value
Untreated	Control	7	3.14	0.63		11.3	3.87		0.051	0.009		-	-	-	-	-	-	-	-	-
	OVX	5	3.04	0.52		8.88	5.18		0.064	0.009		-	-	-	-	-	-	-	-	-
BMP	Control	6 - 7	2.84	0.46		21.0	7.27		0.059	0.006		53.2	21.8		17.2	2.13		274	36	
	OVX	6 - 7	2.61	0.58		14.1	4.03		0.063	0.017		43.2	23.5		16.8	1.19		261	49	
BMP + ZA	Control	5 - 7	3.17	0.37		14.0	7.66		0.057	0.012		41.4	20.0		17.3	1.23		310	43	
	OVX	5 - 7	2.86	0.42		18.0	9.30		0.055	0.009		47.5	14.3		17.2	0.79		298	38	

Supplementary Table 7. Comparison of Treatments: Comparisons of inner callus regions are presented between untreated, BMP-7 and BMP-7 + ZA treatments within either the healthy or the osteoporotic groups.
(***** $p < 0.05$, ****** $p < 0.01$, ******* $p < 0.001$ when compared to Untreated femurs, and
o $p < 0.05$, **oo** $p < 0.01$, **ooo** $p < 0.001$ when compared to BMP only treated femurs).

Control (Nanostructural Parameters)

		Crystal Thickness (Å)			Predominant Orientation (degrees)			Degree of Orientation			D 002 - Crystal Length (Å)			D 310 - Crystal Width (Å)			Crystal Volume (nm^3)		
**Treatment**	**N**	Mean	SD	p-value	Mean	SD	p-value	Mean	SD	p-value	Mean	SD	p-value	Mean	SD	p-value	Mean	SD	p-value
Untreated	7	21.5	1.23		-33.2	6.1		0.59	0.006		-	-	-	-	-	-	-	-	-
BMP	6 - 7	22.3	0.92		-2.7	17.1	**	0.58	0.009	*	346.9	2.05		139.2	2.04		1093	62	
BMP + ZA	5 - 7	21.8	0.89		-13.4	26.9		0.57	0.008	*** o	346.3	2.67		140.7	2.12		1077	49	

Control (Nanostructural Parameters - Heterogeneity)

		Crystal Thickness (Å)			Predominant Orientation (degrees)			Degree of Orientation			D 002 - Crystal Length (Å)			D 310 - Crystal Width (Å)			Crystal Volume (nm^3)		
**Treatment**	**N**	Mean	SD	p-value	Mean	SD	p-value	Mean	SD	p-value	Mean	SD	p-value	Mean	SD	p-value	Mean	SD	p-value
Untreated	7	2.30	0.34		51.9	6.09		0.08	0.013		-	-	-	-	-	-	-	-	-
BMP	6 - 7	2.90	0.68		66.0	9.13	*	0.05	0.006	**	11.8	1.65		23.0	1.11		259	45	
BMP + ZA	5 - 7	2.16	0.39	o	57.4	14.8		0.04	0.004	** o	9.46	1.15	(Ω)	21.8	0.50		221	11	

OVX (Nanostructural Parameters)

		Crystal Thickness (Å)			Predominant Orientation (degrees)			Degree of Orientation			D 002 - Crystal Length (Å)			D 310 - Crystal Width (Å)			Crystal Volume (nm^3)		
**Treatment**	**N**	Mean	SD	p-value	Mean	SD	p-value	Mean	SD	p-value	Mean	SD	p-value	Mean	SD	p-value	Mean	SD	p-value
Untreated	5	22.8	1.14		-25.9	15.7		0.60	0.009		-	-	-	-	-	-	-	-	-
BMP	6 - 7	22.6	1.85		-14.1	28.6		0.59	0.005	*	350.0	3.87		141.3	3.69		1141	134	
BMP + ZA	5 - 7	22.4	1.22		0.48	24.94		0.58	0.015	** o	349.4	4.74		140.7	4.61		1100	111	

OVX (Nanostructural Parameters - Heterogeneity)

		Crystal Thickness (Å)			Predominant Orientation (degrees)			Degree of Orientation			D 002 - Crystal Length (Å)			D 310 - Crystal Width (Å)			Crystal Volume (nm^3)		
**Treatment**	**N**	Mean	SD	p-value	Mean	SD	p-value	Mean	SD	p-value	Mean	SD	p-value	Mean	SD	p-value	Mean	SD	p-value
Untreated	5	2.31	0.23		44.3	4.09		0.07	0.015		-	-	-	-	-	-	-	-	-
BMP	6 - 7	3.83	0.69	**	54.7	13.6		0.05	0.007	*	17.5	4.00		24.5	1.66		317	29	
BMP + ZA	5 - 7	2.39	0.74	oo	64.65	7.40	**	0.04	0.006	** o	11.4	3.49	o	23.0	1.83		249	46	(Ω)

(Ω) $p = 0.052$ when compared to *BMP*

Supplementary Figure 1. Callus – Cortex Comparisons

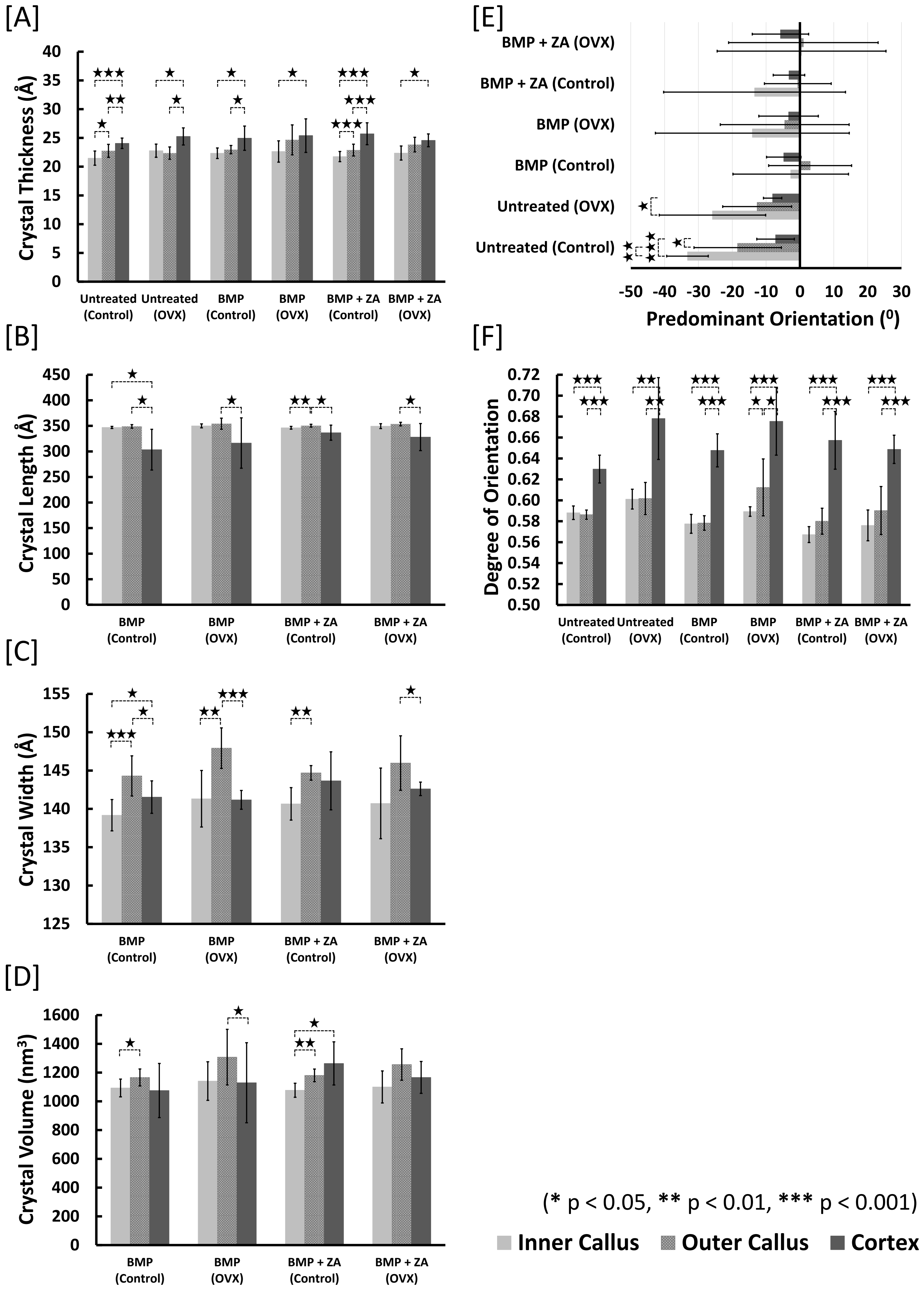

Mathavan et al. (2017)

Supplementary Figure 2. Callus – Cortex Comparisons: Heterogeneity

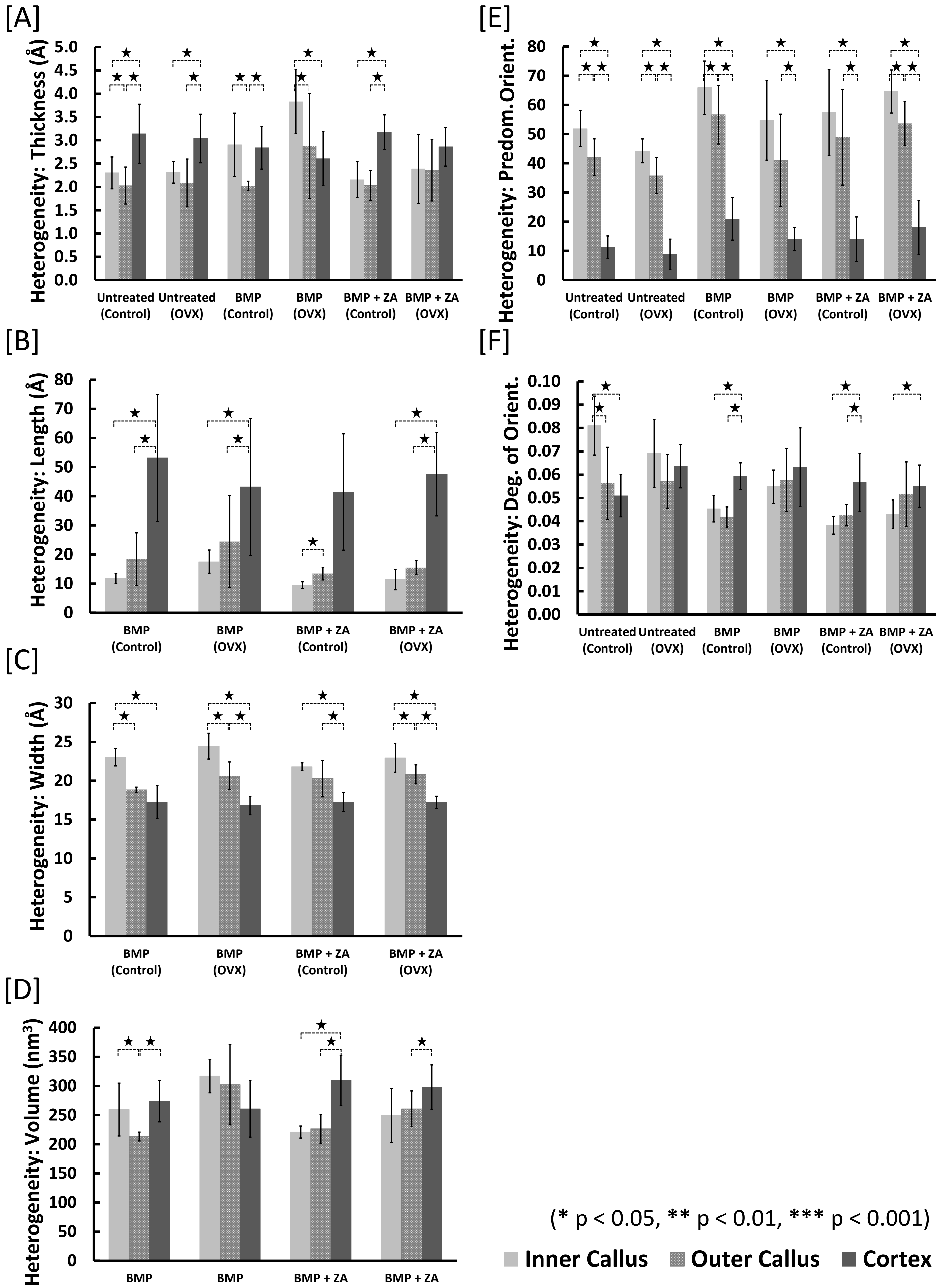

Mathavan et al. (2017)
